# Contribution of transcranial oscillatory stimulation to research on neural networks: an emphasis on hippocampo-neocortical rhythms

**DOI:** 10.3389/fnhum.2013.00614

**Published:** 2013-09-26

**Authors:** Lisa Marshall, Sonja Binder

**Affiliations:** ^1^Department of Neuroendocrinology, University of LübeckLübeck, Germany; ^2^Graduate School for Computing in Medicine and Life Sciences, University of LübeckLübeck, Germany

**Keywords:** tACS, tDCS, sleep, memory, learning, brain rhythms

## Abstract

EEG rhythms reflect the synchronized activity of underlying biological neuronal network oscillations, and certain predominant frequencies are typically linked to certain behavioral states. For instance, slow wave activity characterized by sleep slow oscillation (SO) emerges normally during slow-wave sleep (SWS). In this mini-review we will first give a background leading up to the present day association between specific oscillations and their functional relevance for learning and memory consolidation. Following, some principles on oscillatory activity are summarized and finally results of studies employing slowly oscillating transcranial electric stimulation are given. We underscore that oscillatory transcranial electric stimulation presents a tool to study principles of cortical network function.

The concept that oscillatory brain electric activity—as measured in the EEG or as local field potentials—is more than just an epiphenomenon and can directly impact biological neuronal network activity has existed for some time (Buser and Rougeul-Buser, 1995; Jefferys, 1995; Vigmond et al., 1997). An upsurge of interest in modulating oscillatory activity by applying oscillatory weak electric currents results on the one hand from the recent accumulation of studies on the functional efficiency of applied oscillatory weak electric fields and currents in modulating EEG, local field potentials and neuronal firing rates (reviewed in Weiss and Faber, 2010; Herrmann et al., 2013). On the other hand, the interest in neuronal oscillations and their behavioral relevance has become of increasing interest in the last decade, resulting mainly from studies indicating that precise timing of neuronal activity within oscillatory neuronal networks is essential for information coding and that network oscillations can be a mode of communication between distinct neuronal groups and across brain structures (e.g., Buzsáki and Draguhn, 2004; Fujisawa and Buzsáki, 2011; Hyman et al., 2011; Maris et al., 2011). The study on effects of (transcranially) applied weak oscillatory electric currents is therefore of at least three-fold importance: firstly, as a non-invasive tool for modulating endogenous bioelectric activity, and thus with therapeutic potential; secondly, for investigating the dependence of behavior on brain oscillatory activity; and thirdly, as a tool to study principles of cortical network function.

This mini-review focuses on effects of oscillatory transcranial electric stimulation in particular for learning and for the consolidation of hippocampus-dependent memory. First, an introduction leading up to present concepts and questions on hippocampus-dependent memory consolidation is given. Then we discuss correlates of brain electric activity, cellular and network dynamics. In the second part, features of neuronal and network activity are pointed out which we find relevant to consider when attempting to employ oscillatory stimulation as a tool to study cortical network function.

An association between the hippocampus and memory was established from findings on memory performance in relation to temporal lobe lesions in monkeys (Brown and Schäfer, 1888), hippocampal atrophy (Bechterew, 1900), reports on memory flash backs with hippocampal stimulation (Penfield, 1974), and from reports in the mid-twentieth century differentiating anterograde and retrograde amnesia following well-defined hippocampal lesions as in the case of H.M., the probably most well-known amnestic patient in the history of neuroscientific memory research (Scoville and Milner, 1957). Concepts for neurophysiological memory trace formation, two stage models of memory stage formation, emerged, within which information is transferred to the long term memory store, the neocortex, via hippocampo-cortico connections during the hippocampal sharp wave ripple (SWR) events of slow wave sleep (SWS; Marr, 1970, 1971; Buzsáki, 1989). Later developments of the two stage model aimed to integrate the mechanism of long term potentiation (LTP) in the normal brain (Buzsáki, 1989). It suggested that neuronal firing patterns during hippocampal sharp waves must be the most favorable conditions for enhancement of synaptic plasticity, as SWRs produce powerful synchronization within the pathways connecting the hippocampus to the neocortex (Chrobak and Buzsáki, 1996). The model furthermore incorporated the relevance of behavior and state-dependent changes for defining neuronal patterns (Buzsáki et al., 1987; Buzsáki, 1989).

This concept in which hippocampal theta activity during exploratory behavior in rats supported memory trace formation led to renewed interest in hippocampal place cells (O’Keefe and Dostrovsky, 1971; O’Keefe and Recce, 1993). Subsequent discovery of spatially selective firing of hippocampal place cells in regard to tasks involving spatial memory was the impetus for many investigations on post-experience hippocampal spatiotemporal activity patterns, i.e., reactivation, mostly during SWS (Pavlides and Winson, 1989; Wilson and McNaughton, 1994; Skaggs and McNaughton, 1996; Nadasdy et al., 1999; Hirase et al., 2001; Lee and Wilson, 2002), but also during rapid eye-movement (REM) sleep (Louie and Wilson, 2001); for comprehensive reviews see, Buhry et al. (2011) and Sadowski et al. (2011). Experience-dependent reactivation in sleep was also shown in humans (Rasch et al., 2007; Oudiette and Paller, 2013).

A hallmark of SWS is the endogenous cortical slow oscillations (SO), which coordinates not only thalamo-cortical sleep spindle activity, but also hippocampal SWRs (Timofeev and Steriade, 1996; Isomura et al., 2006; Mölle et al., 2006), slow field potentials with superimposed fast ripple oscillations closely associated with memory consolidation (Fogel and Smith, 2011; Girardeau and Zugaro, 2011). The sleep SO with its coordinating function plays a crucial role in sleep-dependent memory consolidation, specifically for cortico-hippocampal communication (Marshall and Born, 2007). A schematic depiction of these supposed mechanisms is given in Figure 1.

**Figure 1:**
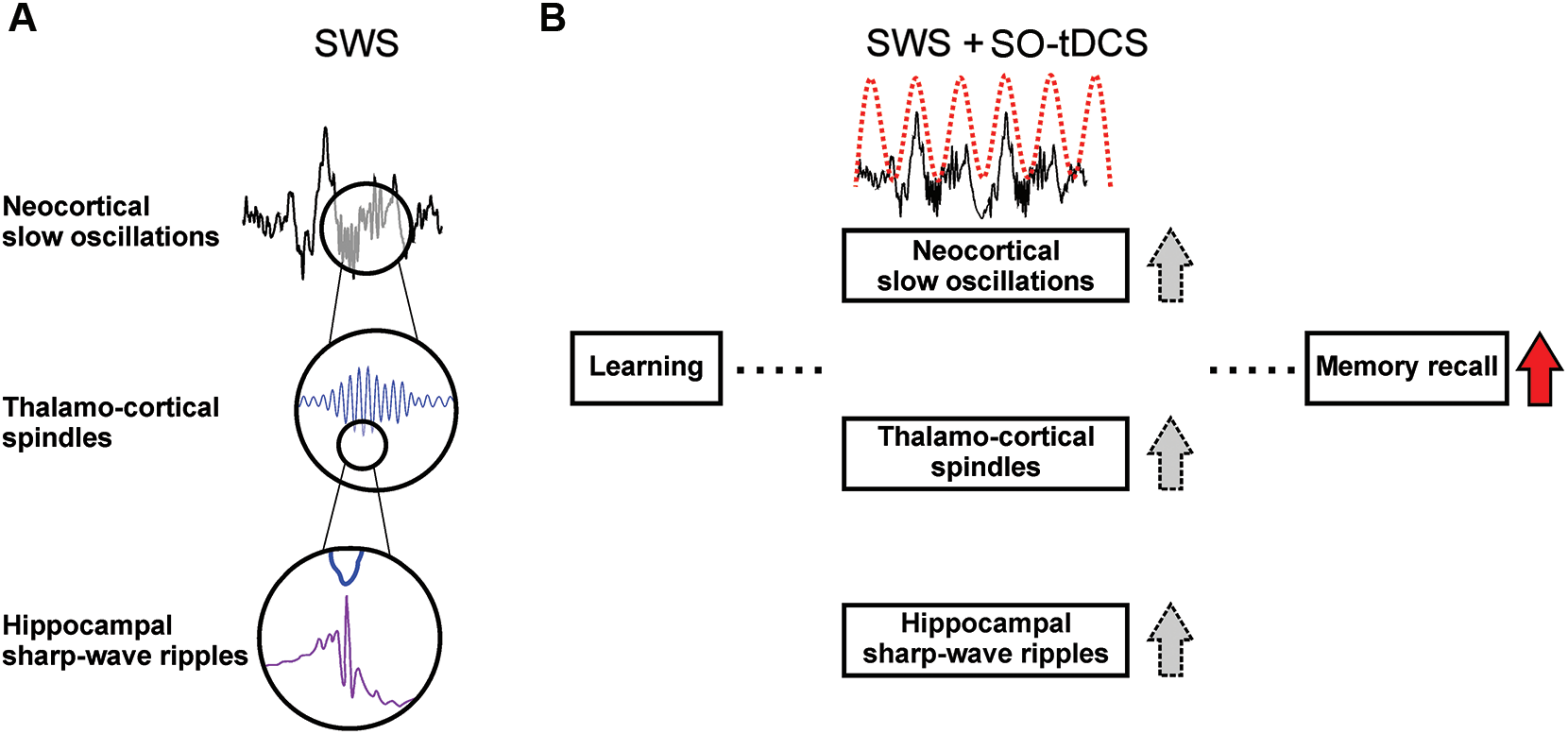
**Sleep-associated brain oscillations relevant for memory consolidation and supposed effects of SO-tDCS (slow oscillatory transcranial direct current stimulation).** **(A)** Temporal relation of SO, sleep spindles and hippocampal SWRs. Sleep spindles and hippocampal SWRs occur preferentially within the Up-state of the SO (Isomura et al., 2006; Mölle et al., 2009). SWRs are temporally coupled to spindles, with individual SWRs nesting into the troughs of spindles (Siapas and Wilson, 1998; Wierzynski et al., 2009; Clemens et al., 2011). Pre-sleep learning enhances activity of and coherence between these oscillations (Mölle et al., 2009), and it is assumed that the interplay of these oscillations subserve the communication between hippocampus and neocortex (Sirota et al., 2003) and therefore the transfer of hippocampus-dependent memory traces from the hippocampal short-term-store to the neocortical long-term store (for review see Marshall and Born, 2007). **(B)** SO-tDCS is assumed to enhance endogenous SO activity, and thus improve the consolidation of memory. It was shown that SO-tDCS enhances post-stimulation power of EEG SO and spindle activity as well as memory consolidation in a hippocampus-dependent task (Marshall et al., 2006). A simultaneous enhancement of these rhythms and SWRs during SO-tDCS yet needs to be shown.

Outstanding experimental support at the level of cell-pairs for the relevance of SWRs for hippocampo-to-neocortical information transfer was given by Wierzynski et al. (2009). During SWRs of SWS, but not during REM sleep, cell pairs showed strong correlations with firing of CA1 hippocampal cells preceding that of prefrontal cell. A functional synaptic connection between hippocampus and prefrontal cortex (PFC) has also been indicated by prefrontal phase locking to hippocampal units during hippocampal theta oscillations while performing a task (Siapas and Wilson, 1998; Hyman et al., 2005). Together these studies indicate nicely that the same neuromorphological structures and pathways are differentially activated dependent on global brain state, i.e., sleep or active task performance. Most importantly, temporally coordinated hippocampal and PFC activity has been most frequently characterized in association with population level activity (Siapas and Wilson, 1998; Sirota et al., 2003; Isomura et al., 2006; Mölle et al., 2006; Peyrache et al., 2011). Aside from being technically more easily obtained than paired single cell recordings, population activity can contain different and vastly more complex information than obtained from single cell recordings (Kopell et al., 2010; Wallace et al., 2011). Coherent firing patterns and enhanced synchronization of rodents’ hippocampal and prefrontal activity has been associated with enhanced memory performance (Benchenane et al., 2010; Fell and Axmacher, 2011; Kim et al., 2011). For instance, Hyman et al. (2010) showed entrainment of medial PFC to the hippocampal theta rhythm correlated with successful performance in a working memory task. Based on these findings it has been suggested that oscillations regulate communication between the hippocampus and medial PFC (Benchenane et al., 2011; Colgin, 2013). However the rules underlying this oscillatory communication, in fact even the rules regarding the relationship of single cells to network activity as well as the interplay between intrinsic properties of the neuron and its inputs, are matters of ongoing research (Akam and Kullmann, 2012).

In the following we point out some essential principles of brain rhythms which indicate how studies employing transcranial weak oscillatory currents can contribute to understanding cortical network function.

Single neurons involved in oscillatory activity do not necessarily fire once per cycle, nor even with the frequency of the network oscillation, but properties of neurons matter with regard to determining collective network synchrony (Jacobs et al., 2007; Csercsa et al., 2010; Wang, 2010). One intrinsic neuronal property relevant for cellular responsiveness and therefore ultimately influencing resultant network activity is preferred resonant frequency. At the single cell level, neuronal resonance typically requires a combination of active and passive properties of a neuron, i.e., passive membrane properties functioning as a low pass filter and voltage-gated active channels which give rise to high pass filtering (Hutcheon and Yarom, 2000; Wang, 2010; Yoshida et al., 2011). Pyramidal neurons in the neocortex can have two resonances which occur at different membrane potential levels (Hutcheon and Yarom, 2000) and neurons of different brain regions have been shown to phase-lock to oscillations at multiple frequencies (Jacobs et al., 2007). Supra- and subthreshold noise, in part arising from neuromodulatory activity, can furthermore significantly affect the interplay between intrinsic properties of the neuron, its inputs and oscillations at network level (Hutcheon and Yarom, 2000; Richardson et al., 2003; Jacobson et al., 2005; Giocomo and Hasselmo, 2007; Wang, 2010; Heys and Hasselmo, 2012).

At the network level, the application of weak oscillatory currents is most effective at the resonance frequency of the network, characterized by the presence of an Arnold’s tongue (i.e., preferred enhancement occurs at this resonance frequency at weak amplitude of the applied current; Ali et al., 2013). Transcranial weak oscillatory currents most commonly induce enhanced EEG activity at the frequency of the applied current. This has been shown and modeled for currents applied at gamma (Strüber et al., 2013), mu/beta (Pogosyan et al., 2009), alpha (Zaehle et al., 2010; Neuling et al., 2012; Merlet et al., 2013), theta (Marshall et al., 2011) and SO’s, the latter in human subjects (Marshall et al., 2006) as well as in animal and in slice experiments (Fröhlich and McCormick, 2010; Ozen et al., 2010). We will focus the below discussion on SO-tDCS, which refers to any stimulation of the same frequency as the endogenous sleep SO (ca. 0.75 Hz in humans), and has a direct current (DC) bias. Another term used so far is transcranial slow oscillation stimulation (tSOS). Precise stimulation parameters (amplitude, duration, shape of the periodic signal and on-/off-set of the oscillatory train) may however vary between experiments. Which effects most of these variables have are yet unclear (see e.g., Groppa et al., 2010, who compared effects of similarly parameterized constant and SO-tDCS).

The mechanisms and prerequisites responsible for resonant EEG activity induced by transcranial weak oscillatory stimulation are still in need of further research. While it is in line with theoretical concepts described above and in Figure 1 that SO-tDCS over the dorso-lateral PFC during SWS in humans enhanced EEG power both in the SO and spindle frequency ranges (Marshall et al., 2006), effects of SO-tDCS during waking are more difficult to reconcile. In waking SO-tDCS enhanced SO’s locally as well as widespread theta activity (4–8 Hz), but not centro-parietal beta activity (Kirov et al., 2009). Was this theta enhancement, also shown to enhance learning, i.e., of functional relevance, only observed because theta was a predominant brain rhythm at this time? Or could EEG theta arise from interactions with specific properties of cellular resonance and/or recurrent network activity? Associations between slow wave and theta band activity exist at many levels, for instance theta nesting in delta activity (Laktos et al., 2005; Carracedo et al., 2013) and parallel modulations in ontogenetic development (Campbell and Feinberg, 2009). Furthermore, similar mechanisms, namely balanced recurrent excitatory and inhibitory activity, have been suggested to underlie the persistent activity during the SO UP state and working memory, the latter being characterized by theta oscillatory activity (McCormick et al., 2003; Reato et al., 2010). However, information on brain state-dependent network dynamics of the interaction between rhythms is still scarce.

The variability in results we and others have observed employing SO-tDCS (e.g., Eggert et al., 2013, who were unable to replicate the results of Marshall et al., 2006, in elderly subjects; Göder et al., 2013, who reported less forgetfulness in schizophrenic patients after stimulation) may in part be inherent to the system. For instance, two studies on SO-tDCS during sleep in healthy individuals showed different results regarding faster rhythms. SO-tDCS during an afternoon nap did not modify spindle power, but did enhance wide-band beta activity as compared to sham (Antonenko et al., 2013). The nap-study differed however in behavioral and temporal parameters from the former, e.g., there was no pre-sleep learning and sleep occurred during a different time of day. Thus not only did experience-dependent features of the neuronal networks differ, but also circadian factors and sleep propensity (such as neuromodulators; Vittoz and Berridge, 2006; Morris et al., 2012; Schmitt et al., 2012). Considering transcranial weak oscillatory stimulation affects subthreshold activity (cp. Reato et al., 2013a, this issue), it is well conceivable that any of the above factors affected single cell and cortical network properties. By virtue of its primary effect on cortical networks we hypothesize that SO-tDCS modifies the efficiency of hippcampo-to-neocortical activity.

Finally, up until now only rather short term effects have been considered, yet memory can improve across days with repeated learning. Constant tDCS has been shown to modify plasticity related products (Fritsch et al., 2010; Stagg and Nitsche, 2011). Long term modifications in oscillatory neuronal activity have to our knowledge only been reported up to 30 min in a state-dependent manner for alpha-activity following transcranial alternating current stimulation (tACS) at individual alpha frequency following stimulation (Neuling et al., 2013), and a putative role of spike-time dependent plasticity for after-effects of alpha-tACS were tested so far in simulations only (Zaehle et al., 2010). At the network level, responsiveness to acute SO-tDCS in rats appears to be affected after about 1 week of daily stimulation subsequent to learning on a spatial task (Binder et al., 2012). Although we can as yet not ascertain that learning or plastic changes in the cortical network occurred throughout the above experiment, the long-term implications of the study are that network “learning” can be induced and the dynamics and mechanisms of this process could in future be measured in detail.

Findings that the most consistent effect of SO-tDCS during SWS is on the endogenous sleep slow oscillatory rhythm implies that this oscillation of neocortical origin was primarily impacted by SO-tDCS, and causally affected memory consolidation and learning. But, selective activation and deactivation of other brain structures within the circuit, in combination with other methods, e.g., optogenetics, is furthermore required to highlight the specific function of the neocortical network for memory consolidation. Furthermore, the differential results of transcranial weak oscillatory stimulation due to brain state point out the necessity, as technical capabilities develop, to consider this state-dependency in research approaches investigating local networks and neuronal properties, e.g., by mimicking different brain states in slice preparations. Finally, development and extension of computational network models can help guide systematic studies on transcranial weak oscillatory stimulation investigating coupled rhythms (e.g., Reato et al., 2013b).

## Conflict of interest statement

The authors declare that the research was conducted in the absence of any commercial or financial relationships that could be construed as a potential conflict of interest.
